# Introducing *Life Science Alliance*

**DOI:** 10.26508/lsa.201700001

**Published:** 2017-12-07

**Authors:** Andrea Leibfried

**Affiliations:** Executive Editor, *Life Science Alliance*, Heidelberg, Germany

## Abstract

A new open-access journal by leading not-for-profit research institutions focusing on quality and reliability with a rigorous, transparent, and academically driven peer-review process.

***Life Science Alliance*, a new open access journal published jointly by three leading not-for-profit research institutions, focuses on quality and reliability based on a rigorous, transparent, and academically driven peer review process. *Life Science Alliance* speeds up the publication of important research submitted to any of the journals of the alliance partners.**

The aim of *Life Science Alliance* is to make publishing more efficient, constructive, and fair. The not-for-profit venture is built on a partnership between EMBO Press, Rockefeller University Press, and Cold Spring Harbor Laboratory Press.

**Figure 1:**
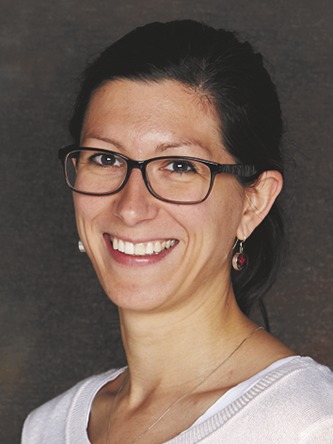


## An open access journal with inclusive scope

*Life Science Alliance* selects valuable results in all areas of the life sciences and biomedical research. The journal publishes original research, methods, and resource articles. *Life Science Alliance* will have a peer review process that is as thorough as the other journals in the alliance in order to publish rigorously executed research papers on new results of particular value to the research community, but without emphasis on conceptual novelty. We also welcome important confirmatory results, descriptive or negative data, resources such as datasets, tools, and screens, as well as new methods.

## Rapid, low friction publication without futile rounds of peer review

*Life Science Alliance* has no format restrictions and minimal requirements for initial submission. The partner journals^1^ encourage authors to get their work published more quickly and with minimal friction by offering to transfer selected manuscripts to *Life Science Alliance*. Our aim is to provide an alternative to (re)submission to multiple journals and assessment by different sets of referees to speed the time to publication and reduce the burden placed both on authors and referees.

When it is in the interest of the authors, the editors of partner journals invite transfers to *Life Science Alliance* before or after peer review. Importantly, this happens after a careful consultation between the editors of both journals, so that authors are given a clear commitment and guidance to the next steps. At all times, the authors retain full control of their work and where they intend to publish—they can opt out of the consultation or forgo the invitation to transfer.

## A home for every compelling paper

We see it as our mission to give scientists the opportunity to publish valuable papers with only one set of referee reports and no more than one round of major revision. This applies equally to manuscripts submitted directly to *Life Science Alliance* and those transferred from EMBO Press, Rockefeller University Press, or Cold Spring Harbor Laboratory Press journals. Working with the referee reports from a previous round of peer review allows us to make a firm commitment toward publication in *Life Science Alliance* to authors upfront and, importantly, to restrict revisions to the essentials. Our aim is to prevent repetitive, lengthy rounds of review, avoiding referee exhaustion, author frustration, and slow dissemination of research results.

## A community-driven journal

*Life Science Alliance* is pleased to work in conjunction with academic editors who are leading scientists committed to a community-oriented, constructive publishing process. Julia Cooper, Florent Ginhoux, Sebastian Jessberger, and Michael Overholtzer will shape *Life Science Alliance* and support the goal of the three institutional alliance partners in giving authors the choice to publish high quality work in a not-for-profit, inclusive setting. 

*Life Science Alliance*’s editorial process is efficient and provides authors with the best editorial experience: dedicated professional scientific editors and research-active academic experts drive the process with advice from a committed, young academic board. This ensures community-driven editorial decisions with the aim to publish research of explicit value to the respective fields. The collaborative editorial process ensures timely, balanced decisions across all subject categories.

Our aim for *Life Science Alliance* is to enable the scientific community to disseminate notable results in a not-for-profit, community-oriented journal. We commit to actively involve younger scientists, including experienced postdocs as reviewers, in order to create a forward-looking, constructive publishing platform.

## Transparent and balanced decision-making and credit to referees

*Life Science Alliance* adopts the qualities of the journals already published by the three alliance partners and applies them to direct submissions and transferred manuscripts alike.

*Life Science Alliance* stands not only for a fair and rapid review process but also for transparency. Reviewers are encouraged to comment on each other’s reports to allow for balanced decision-making, and referee reports from *Life Science Alliance* as well as the decision letters of the editors and author responses will be published alongside papers. *Life Science Alliance* will work to give credit to our valued referees by showcasing how they improve the papers and providing reference letters and certificates to referees upon request.

*Life Science Alliance* is fully open access (CC-BY) and encourages publishing source data without restriction (CC-0) for the efficient sharing, discovery, and reuse of research results. *Life Science Alliance* embraces open data and open science principles, including deposition and co-posting with submission to preprint servers.

## 

I hope that you will share our enthusiasm for this unique venture and help *Life Science Alliance*’s aim to catalyze research progress by removing barriers to the dissemination of valuable research findings. Like the alliance partner institutions—EMBO, Rockefeller University, and Cold Spring Harbor Laboratory—*Life Science Alliance* strives to excel. The journal will update and adapt its editorial process and scope to meet community expectations and be at the cutting edge of the different fields. I am eagerly looking forward to sharing our first issue with you alongside all *Life Science Alliance* has to offer you. I welcome you as a reader, ambassador, and author!

## Footnotes

1 EMBO Molecular Medicine, EMBO reports, Genes & Development, Genome Research, Journal of Cell Biology, Journal of Experimental Medicine, Journal of General Physiology, The EMBO Journal, Molecular Systems Biology

